# The evolution from the two stage to the one stage procedure for biofilm based periprosthetic joint infections (PJI)

**DOI:** 10.1016/j.bioflm.2020.100033

**Published:** 2020-08-05

**Authors:** Gerhard E. Maale, John J. Eager, Aniruth Srinivasaraghavan, Daniel Kazemi Mohammadi, Nicole Kennard

**Affiliations:** a Dallas-Ft. Worth Sarcoma Group, 4708 Alliance Blvd, Plano, TX, 75093, United States; b Emory University Hospital System, 1364 Clifton Rd, Atlanta, GA, 30322, United States

**Keywords:** Biofilm One Stage, Two Stage, Periprosthetic Joint Infection (PJI), Calcium Sulfate Hemihydrate Antibiotic Loaded Pellet, Radical Debridement

## Abstract

A definitive consensus on the optimal limb salvage protocol for infected total joints does not currently exist. Popular, is the two-stage revision which calls for the use of an antibiotic loaded spacer followed by a delayed exchange. Our question is whether single-stage revisions for biofilm based infected arthroplasties results in comparable or possibly better patient outcomes as compared to those reported for two-stage revisions. We retrospectively reviewed 500 cases of one-stage revisions for periprosthetic joint infections (PJI) using dual setup with radical debridement, definitive reconstruction with antibiotic loaded cement and implantation of antibiotic calcium sulfate pellets between the years 2005–2017. The revisions included 351 total knees, 122 hips, 2 hip-femur-knees, 13 shoulders, 10 elbows, and 2 shoulder-humerus-elbows. The patient population had a mean follow-up of 60 months (range: 24 months–14 years) and mean patient age of 61 years old, consisting of 250 males and 250 females. Patient comorbidities were reviewed, classified using McPherson’s staging for PJIs, and compared to the Cierny & Mader classification system. Successful treatment was defined as a joint without recurrence of infection, for a minimum of 2 years, and limb preservation. Based on our findings, one-stage revision of PJIs demonstrates at least as good an infection eradication rate as two-stage revision: 88% vs 85% respectively.

## Introduction

The occurrence of deep infection of total joints is a serious complication requiring aggressive management to eradicate infection and salvage the joint. One of the main factors complicating the course of these patients is the formation of microbial aggregates known as biofilms on implants, dead tissues, and foreign bodies [6,13,14,22]. These collections of microorganisms, through a process identified as quorum sensing, have the ability to interact via secretion and detection of diffusible signals. The result of this is the formation of an exopolymer substance and the maturation of what we call biofilm [12]. Conversion of planktonic microorganisms to sessile (biofilm) forms allows the infecting pathogen to avoid destruction by host immune mechanisms and systemic antibiotics [9,16,22]. This is further complicated by high mutation rates and extensive exchanges of genetic material in biofilms which promote antibiotic resistance [39]. The biofilm acts as an adsorbent of phage and plasmid. In fact, as much as 10–15% of the bacterial genome becomes infiltrated with virus [39]. However, the adaptive immune system of bacteria utilize clustered regularly interspaced short palindromic repeats/CRISPR-associated (CRISPR/Cas) 1–3 as a natural defense mechanism against viral infection [19]. This is because the CRISPR/Cas system allows the bacteria’s immune system to recognize and destroy the foreign viral genetic material in a highly adaptable and heritable mechanism [19]. It takes to kill bacterial biofilm log 3 above the minimum inhibitory concentration (MIC) to kill the biofilm with systemic antibiotics [13,26]. Biofilm with systemic antibiotics kills the outer cells, but the inner, low metabolic cells persist (persister cells) [23]. As a result, systemic antibiotics may be used to calm biofilm-related infections down but do not eradicate them. Because of this, surgical excision of the biofilm related infection is the mainstay of treatment.

Surgical revision protocols of PJI, designed to address the nature of these infections, were first introduced over 3 decades ago [2,3,20,27]. The fundamental concepts for treatment of PJI described the necessity for implants, foreign bodies, and cement removal in combination with radical debridement, the process of removing dead soft tissue and bone. This is coupled with the use of antibiotic loaded cement and other molecular carriers along with stabilization of the joint with either a definitive or temporary prosthesis for treatment [13,33,44]. It is upon these principles that today’s limb salvage protocols have evolved. Alternative therapies in the past included resection arthroplasties, fusions, and amputations. Surgeons are now able to take advantage of growing technological innovations and employ aggressive surgical techniques for debridement followed by the restoration of function in patients with PJI.

Over the years, a two-stage procedure has emerged as the “gold standard” of treatment for chronic infection of total joints [3,13,15,27,31,[45], [46]]. In 1993, we first described the two-stage treatment for PJIs using articulating spacers and antibiotic loaded cement at high doses. The exchange interval to the definitive reconstruction was two weeks based on the local bleaching of the antibiotic cement in our study group. The success rate was approximately 85% eradication based on two thousand cases [8]. Others have reported success rates ranging from 60% to 100% with this technique in smaller patient populations, [21,24,32,[34], [37]]. Despite its obvious success and widespread implementation, a two-stage revision is not without drawbacks. The time between stages is commonly associated with impaired mobility, joint stiffness and pain [2,20,38]. Additionally, arthrofibrosis developing between stages can make reimplantation difficult [38,46]. Furthermore, the requirement of multiple surgeries is inevitably associated with increased perioperative morbidity and a protracted hospital course, leading to elevated medical costs [10,17,[36], [37]].

An alternative procedure involves only one operation with immediate exchange of the infected joints. This procedure involves radical debridement requiring a dual setup in the operating room with a dirty and clean side. Additionally, antibiotic loaded cement and antibiotic loaded calcium sulfate hemihydrate pellets are used as local antibiotic carriers. These carriers can get to log 3 MIC locally without systemic levels being detected and allow for the definitive prosthesis replacement. The antibiotic loaded calcium sulfate hemihydrate with 240 ​mg of tobramycin and 500 ​mg of vancomycin per 10 ​cc completely killed Pseudomonas ERC-1, and MRSA using the Center for Biofilm Engineering (CBE) biofilm drip reactor [26]. The eluent had no viable bacteria.

The execution of one-stage surgical intervention involves several key components. Intraoperatively, extensive radical debridement is essential for removal of the biofilm-based infection. This requires an “oncologic-type” of surgical debridement which includes removal of all prosthetic components, dead and reactive tissues, and foreign material such as braided sutures and prosthetic debris [13]. In scenarios where there is inadequate soft tissue, provisions must be made for local and free muscle flaps. It requires mobilization of adjacent vessels and nerves in and around the joint as well as resection of involved dead ligaments and tendons that are infected with biofilm. Our protocol also calls for a 6- week course of postoperative intravenous antibiotics and 4.5 months of oral antibiotics. It is careful adherence to this standardized type of protocol which allowed for specified analysis of results.

Our purpose was to question the effectiveness of a one-stage procedure in controlling total joint infection as compared to the more commonly accepted two-stage approach.

## Materials and methods

### Case identification

Between 2005 and 2017, 500 infected arthroplasties were treated with a one-stage revision. The 500 cases were referred to us with a historical average of 5–7 surgical treatments prior to referral. Almost all the patients referred had known PJI.

### Preoperative evaluation and diagnosis

The presence of infection was determined based on a combination of clinical history/presentation as well as examinations. Evaluation of clinical history/presentation included the identification of elements that characterize PJI such as a draining sinus tract, wound healing problems, bacteremia, and other etiologies described in Table 1. Other indications that were also considered including sudden onset of joint pain, effusion, swelling, calor, or erythema indicative of an acute inflammatory process.

**Table 1:** Criteria for the diagnosis of clinically suspected PJI [35].

	**Infection Unlikely** (all findings present)	**Infection Likely** (at least two positive findings)	**Confirmed Infection** (any positive finding)
**Diagnostic Test**			
Clinical Features	Clear alternative reason for implant dysfunction (fracture, implant breakage, malposition, tumour)	1) Radiological signs of loosening within the first 5 years after implantation. 2) Previous wound healing problems 3) History of Bacteremia 4) Purulence around the prosthesis	Sinus tract with evidence of communication to the joint or visualization of the prosthesis
**Blood Biomarkers**			
C-Reactive Protein		CRP >10 ​mg/L (1 ​mg/dL)	
**Synovial Fluid Cytological Analysis**			
Leukocyte count (cells/μL)	≤1500	>1500	>3000
PMN (%)	≤65	>65	>80
**Synovial fluid Biomarkers**			
Alpha-defensin		Immunoassay ​≥ ​5.2 ​mg/L or Lateral-flow Test positive	
**Microbiology**			
Aspiration Fluid	Culture Negative	Positive culture	Positive culture
Fluid and tissue obtained at surgery	All cultures negative	Single positive culture	≥2 positive samples with the same microorganism
Sonication (CFU/mL)	No growth	>1 ​CFU/mL of an uncommon contaminant	>50 ​CFU/mL of any organism
**Histology**			
	Negative	Presence of ≥5 neutrophils in a single high-power field (400x magnification)	Presence of ≥5 neutrophils per high-power field in ≥5 high-power fields (400x magnification)
			Presence of visible microorganisms on histological sections
**Others**			
Nuclear Imaging	Negative 3-phase Isotope Bone Scan or WBC Scintigraphy	Positive WBC scintigraphy	

**Fig. 1:**
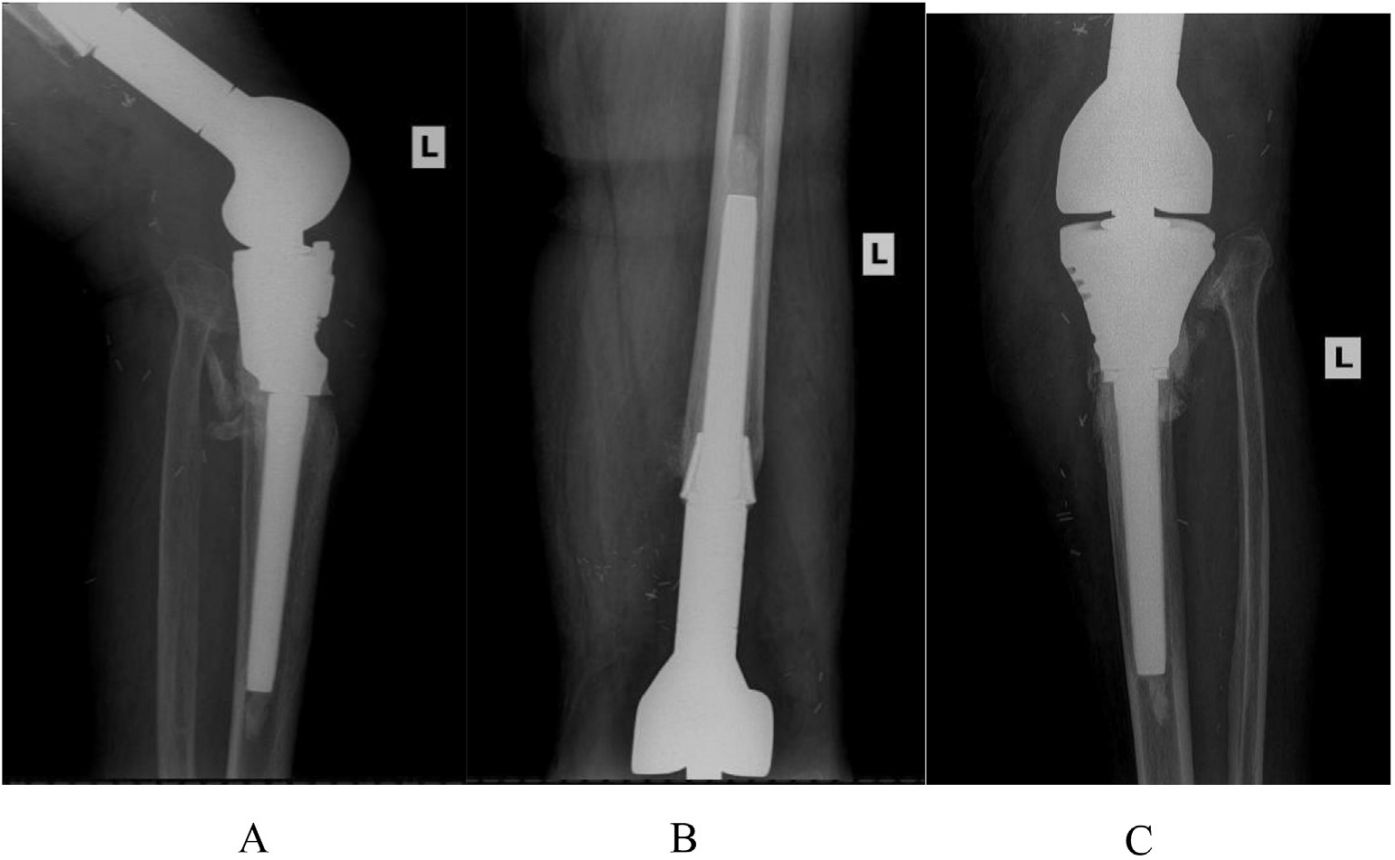
X-rays were taken prior to staging showed a well-fixed tibia and femoral stem. A. AP projection. B. Lateral projection. C. Proximal femur.

**Fig. 2:**
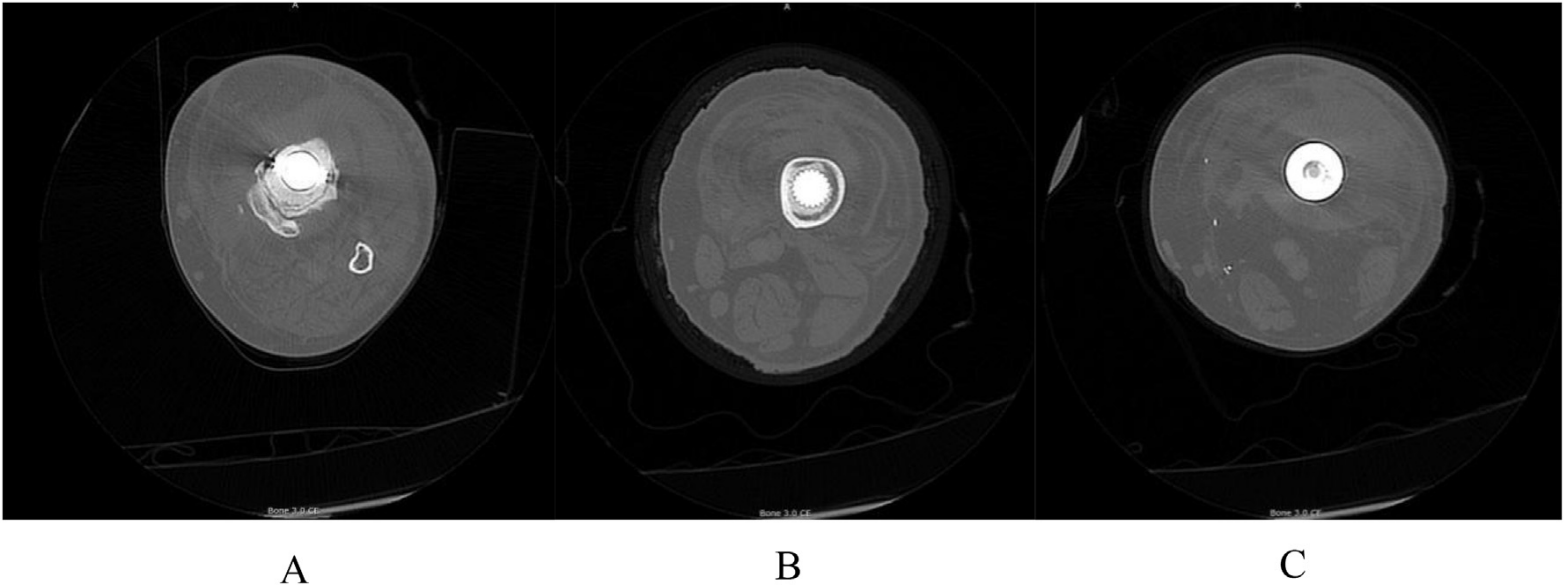
A CT scan provides a three-dimensional look inside the bone, muscle, and fat. Transaxial cuts from distal to proximal sections along the leg (images A-C) are from the same patient. A. Demonstrates increased reactivity around the tibial component. B. Demonstrates a well-fixed femoral component with synovial effusion. C. Demonstrates large effusion adjacent to the femoral component as well as reactive soft tissue.

**Fig. 3:**
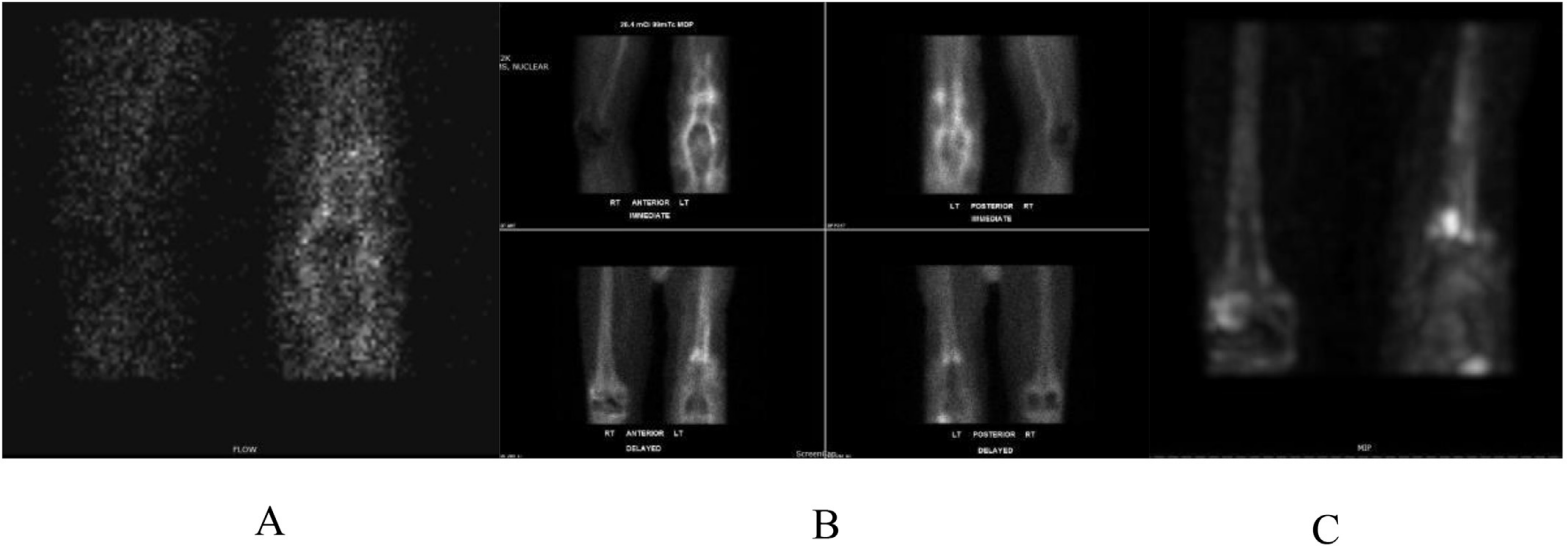
A triple phase bone scan indicates an increased uptake around the prosthetic component in the left knee. A. Early flow phase. B. Late vascular flow phase showing reactive synovitis. C. Delayed uptake shows reaction around femoral component.

**Fig. 4:**
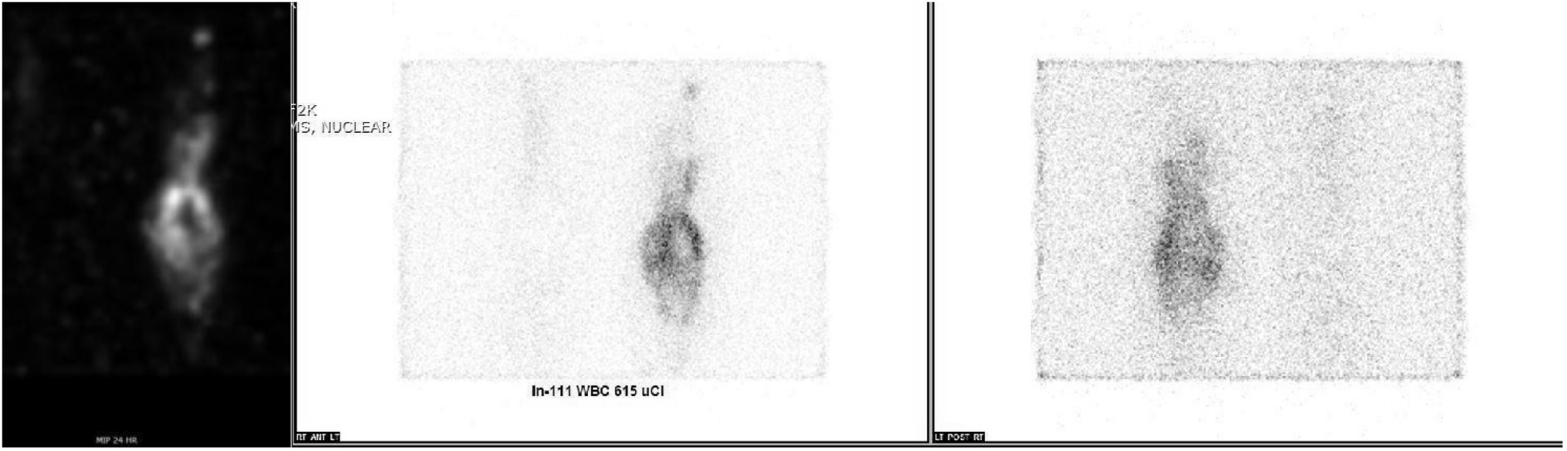
Indium-labeled WBC scan showing increased uptake suggestive of acute inflammation and infection.

Factors such as concomitant drug intake, inflammatory arthropathy, recent surgery or fracture, adverse local tissue reaction (ATLR), and crystal arthropathy were also considered as these may limit the usefulness of certain diagnostic tests [35]. Additionally, patients underwent preoperative labs which included the following: Comprehensive Metabolic Panel (CMP), Complete Blood Count (CBC) with differential, coagulation panel, C-Reactive Protein (CRP), and Erythrocyte Sedimentation Rate (ESR). Patients being considered for surgery also took a HIV and hepatitis screen. A number of studies were taken to localize and diagnose the infection (Fig. 1, Fig. 2, Fig. 3, Fig. 4). The studies included a Tc-99 triple phase bone scan and a CT of the infected joint, and an indium-tagged WBC scan, used to identify the presence of an infectious process in a localized area.

### Clinical staging

Systemic and local compromises were defined by Cierny and Mader and later modified by McPherson et al. for staging classifications of PJI [5,30]. Details of this classification system are provided in Table 2, Table 3 (Cierny and Mader) and Table 4, Table 5 (McPherson). These staging systems account for the acuteness or chronicity of infection, the overall medical and immune health status of the patient, and the local wound compromising factors [[28], [29],^30^]. The efforts of classifying each infection were designed to assist the surgeon in identifying the severity of each case and to choose appropriate treatment regimens [5,6,29,30]. Whenever possible, efforts were undertaken to medically treat amenable comorbidities in order to provide for host optimization prior to surgery. For example, patients who smoked tobacco were required to quit for a period of at least one month prior to surgery. In addition, local compromising factors were addressed for patients that required hyperbaric oxygen (HBO), suction vac, and vascular workup for flaps. The patient had to clear standard preoperative evaluation in addition to evaluation by infectious disease consultants. Patients were also recommended an adequate nutritional diet to ensure optimal recovery post-surgery.

**Table 2:** Cierny and Mader Staging system for long bone osteomyelitis [5,8].

Anatomic Type	Systemic Host Grade
● **Type I** – Medullary osteomyelitis	● **A Host** – Good immune system and delivery
● **Type II** – Superficial osteomyelitis	● **B-Host** – Compromised locally (Bl) or Systemically (Bs)
● **Type III** – Localized osteomyelitis	● Systemic and local compromise (Bls)
● **Type IV**– Diffuse osteomyelitis	● **C-Host** – Requires suppressive or no treatment; minimal disability; treatment worse than disease; not a surgical candidate

**Table 3:** Systemic and local factors in class B hosts [5,8].

Systemic Factors	Local Factors
● Malnutrition	● Chronic lymphedema
● Renal, hepatic failure	● Venous stasis
● Diabetes mellitus	● Major-vessel Compromise
● Chronic hypoxia	● Arteritis
● Immune disease	● Extensive scarring
● Malignancy	● Radiation fibrosis
● Extremes of age	● Small-vessel disease
● Immunosuppression or immune deficiency	● Neuropathy
	● Tobacco abuse (>2 packs/d)

**Table 4:** McPherson Staging Classification for periprosthetic infection^a^ [29].

Anatomic Complexity	Systemic Host Grade	Local Host Grade
● Type I – Early postoperative infection (<4 postoperative weeks)	● A –Uncompromised	● 1 – No local compromise
● Type II – Hematogenous infection (<4 weeks duration)	● B – Compromised; ≤2 compromising factors	● 2 – Compromised ≤2 local compromising factors
● Type III – Late chronic infection (>4 weeks duration)	● C – Significant compromise; ≥3 compromising factors or one of the following:	● 3 – Significant local compromise; ≥3 local compromising factors
	● Absolute neutrophil count <1000	
	● CD4 T cell count <100	
	● Chronic active infection at another site	
	● Dysplasia or neoplasm of the immune system	

a Staging criteria used in the classification of patients with chronic osteomyelitis.

**Table 5:** Compromising host factors^a^.

Systemic Factors	Local Factors
● Immunosuppressive drugs	● Multiple incisions with skin bridges
● Alcoholism	● Active infection present >3 months
● Hypoxia	● Soft tissue loss from prior traumas
● Malignancy	● Subcutaneous abscess >8 ​cm^2^
● Diabetes	● Synovial cutaneous fistula
● Old Age (>80 years)	● Prior periarticular fracture or trauma about a joint
● Chronic active dermatitis or cellulitis	● Prior local irradiation
● Pulmonary insufficiency	● Vascular insufficiency to extremity
● Nicotine use	
● IV drug abuse	
● Chronic indwelling catheter	
● Chronic malnutrition	
● Renal failure requiring dialysis	
● Systemic inflammatory disease	
● Systemic immune compromise	
● Hepatic insufficiency	

a Classification of comorbidities that affect wound healing in the treatment of chronic osteomyelitis, as described by Cierny et al. and McPherson et al.

### Surgical treatment for infection

Surgical intervention involved an open biopsy of joints with frozen-sections that were submitted for H&E preparations. Acute inflammation was defined as greater than 20 neutrophils per high power field with clumping. Specimens were sent for anaerobes, aerobes, AFB, and fungal cultures.

**Fig. 5:**
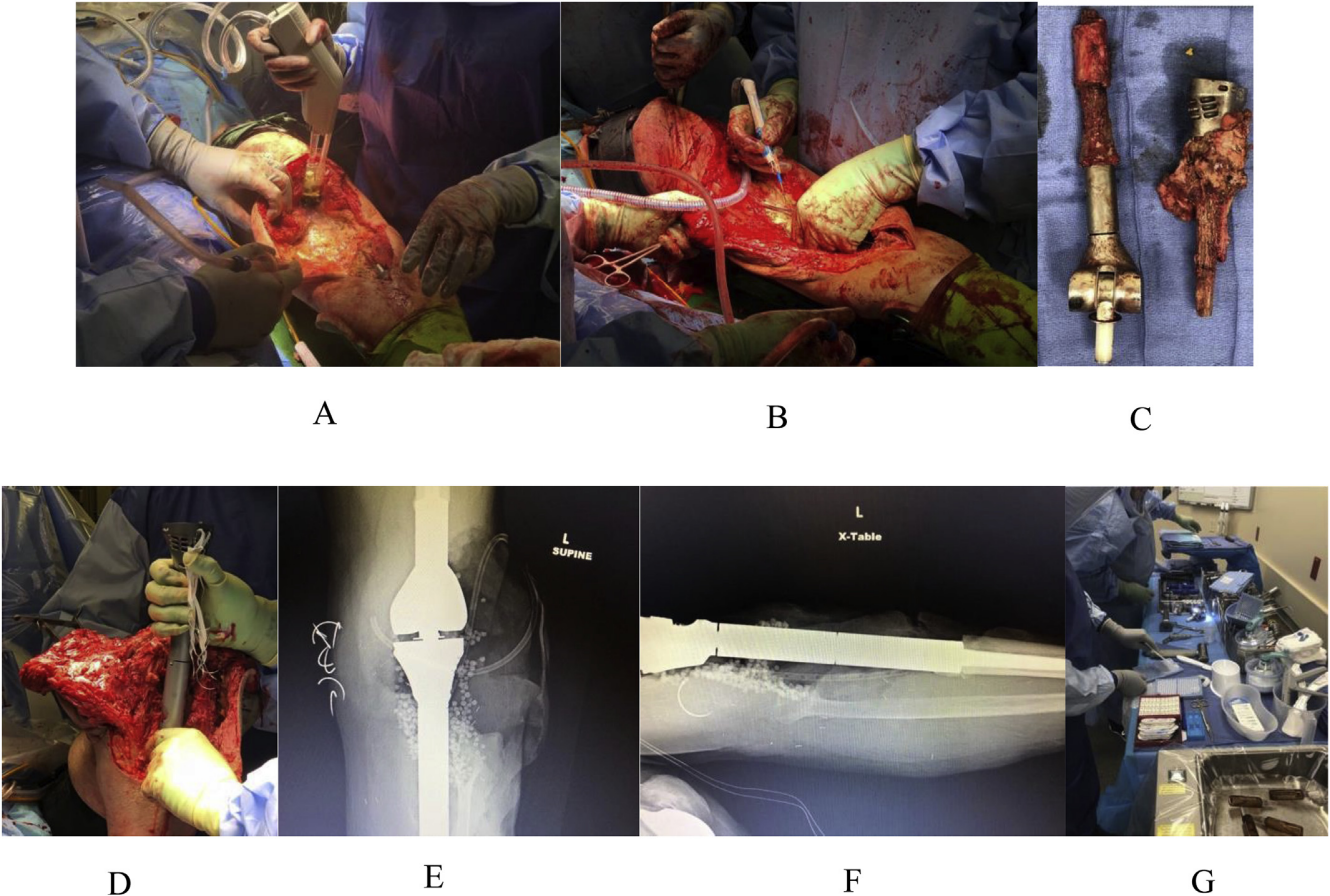
The above images (A–G) show the complete one stage procedure for the same patient. Figures A–C show procedures performed on the dirty side. Figures D–G shows procedure performed on the clean side. A. Radical debridement includes the excision of all devitalized soft tissue and bone, hardware, foreign bodies, and reactive tissues, leaving sometimes massive bone and soft tissue defects. B. Removal of the prior prosthetic component with additional infected bone. C. After completion of radical debridement, the surgical site underwent pulsatile irrigation with 6 L of saline followed by changing of all gowns, gloves, drapes, and instrumentations. D. Preparation of antibiotic loaded calcium sulfate hemihydrate pellets and assembly of the new prosthesis. E. Implantation of sterile tibial prosthetic component on the clean side. F. Postoperative AP view showing implanted antibiotic loaded calcium sulfate hemihydrate pellets around prosthesis. G. Postoperative lateral view showing prosthesis prior to free flap.

Following the biopsy, a radical debridement is performed consisting of removal of all biofilm involved hardware, devitalized soft tissue and bone. The hardware includes the prosthetic component, cement, and foreign bodies such as braided suture material. In addition, the foreign material and dead or damaged wound tissues were resected. Major vessels and nerves around the joint were mobilized during the debridement process. Prosthetic trials were done on the dirty side. This was followed by irrigation with 6 ​L of normal saline via pulsatile flow. Fig. 5A–C shows procedures performed on the dirty side.

After irrigation, gowns, gloves, drapes, and instruments were changed on the clean side, and the wound was reirrigated with another 6 L of saline solution. A definitive reconstruction with new prosthetic components was fixed with antibiotic loaded cement. As a result of the extensive resection of soft tissue, a constrained prosthesis was required [25]. The antibiotic cement contained 2 ​g of Vancomycin and 5 ​g of Fortaz per mixture of Simplex [10,15,18,39]. Following the cementation, antibiotic loaded pellets were placed in dead spaces around the prosthesis at a concentration of 240 ​mg of liquid tobramycin and 500 ​mg of vancomycin powder per 10 ​cc of the hemihydrate [1,5,7,46]. Fig. 5D–G shows procedure performed on the clean side.

**Fig. 6:**
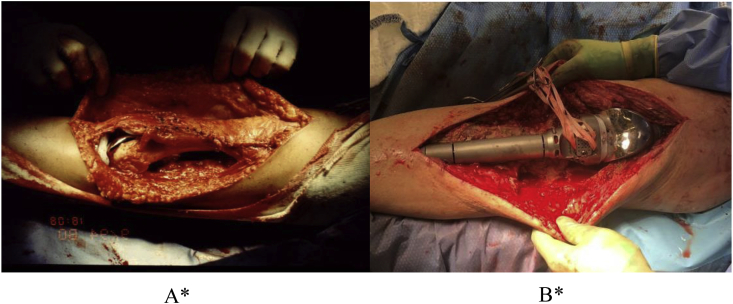
Due to the extent of debridement undertaken, it was necessary to provide for soft tissue coverage. Muscle flaps were applied as needed in order to provide adequate soft tissue coverage. A. Inadequate soft tissue coverage necessitating intervention with flap. B. Subcutaneous tissue is inadequate for coverage in these scenarios. In this case, it requires a local muscle flap. ∗Images A and B represent different patients undergoing the same procedure.

Due to the extent of debridement undertaken, it was necessary to make provisions for necessary soft tissue coverage (Fig. 6). Free or local muscle flaps were applied as needed in order to provide adequate soft tissue coverage over the prosthesis [29].

### Post-operative antibiotic management

All patients received a 6-week course of intravenously administered antibiotics with 4.5 months of oral antibiotics following surgery based on prior cultures. For patients that presented with chronic lymphedema, venous stasis, or other severe local compromising factors, provisions were made for lifetime suppression with antibiotics. In cases where cultures failed to reveal an infecting organism despite clinical evidence of an ongoing infection, antibiotic selection was directed at organisms commonly implicated in PJI. A combination of antibiotics was given to patients in order to control both gram negative and gram positive organisms, as well as fungal organisms in patients with open draining sinus tracts.

### Clinical analysis

Patients were followed clinically and radiographically. Patients followed up two weeks after the date of surgery with an x-ray. From there, patients followed up with x-rays at 6 weeks, 3 months, 6 months with lab results, including CBC, sedimentation rate, and C-reactive protein, and finally yearly follow-ups. Patients were followed up for at least 2 years.

## Results

All patients presented with multiple comorbidities and were subsequently staged as illustrated in Table 3. The patient population consisted of 250 females and 250 males with an average age of 61 years (range: 23–88) with an average follow-up of 60 months (range: 24 months–14 years). There were 351 knees, 122 hips, 2 hip-femur-knee, 13 shoulders, 10 elbows, and 2 shoulder-humerus- elbows.

Organism retrieval rate was about 34.4%. The incidence of culture negatives (65.6%). MRSA and MSSA had the highest culture positivity rate in the infected patient population (10.2% and 4.6% respectively). In the cases where patients were culture negative, diagnosis of infection was made through a combination of clinical observations, histology, radiological imaging, and laboratory markers. Table 6 breaks down incidence rates by types of organisms.

**Table 6:** Organism incidence.

Organism	N	Percent
MSSA	21	4.2
MRSA	53	10.6
Cellulitis	9	1.8
Hepatitis C virus	3	0.6
*Staphylococcus epidermidis*	16	3.2
*Staphylococcus epidermidis* (methicillin resistant)	1	0.2
*Staphylococcus hominis*	1	0.2
*Staphylococcus lugdunensis*	1	0.2
Unidentified *Staphylococci* species	15	3.0
*Streptococci* Group D	6	1.2
*Streptococcus viridans*	1	0.2
*Stenotrophomonas maltophilia*	1	0.2
Yeast Infection	1	0.2
*Propionibacteria*	1	0.2
*Enterococcus faecalis*	1	0.2
*Corynebacteria*	5	1
VRE	2	0.4
*Enterobacter aerogenes*	1	0.2
*Pseudomonas aeruginosa*	2	0.4
*E. coli*	3	0.6
*Klebsiella oxytoca*	1	0.4
*Candida albicans*	4	0.8
*Candida parapsilosis*	1	0.2
Unidentified *Candida* species	5	1
Acid fast Bacilli	2	0.4
Unidentified Gram-Positive Cocci	1	0.2
Unidentified aerobic organism	1	0.2
*Salmonellae*	1	0.2
*E. cloacae*	1	0.2
Polyclonal^a^	11	2.2
Culture negative	328	65.6

a Incidence of more than one infecting organism was classified as polyclonal.

**Fig. 7:**
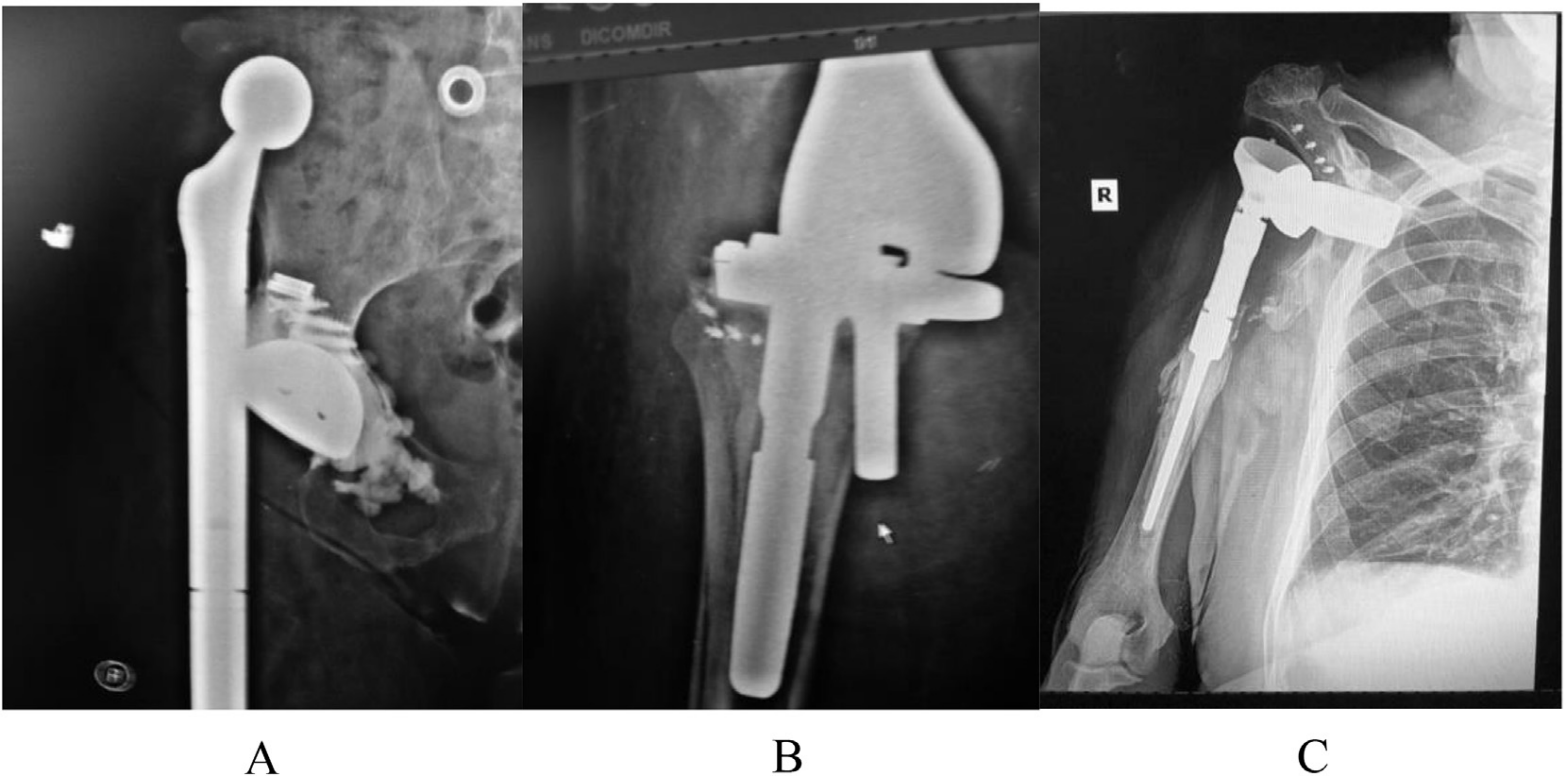
Dislocation is an adverse event and one of the main reasons for recurrence of infection. One of the primary reasons for dislocations is a lack of soft tissue. A. Dislocated constrained total hip. B. Dislocated total knee. C. Dislocated total shoulder.

60 out of 500, or 12% of patients recurred with infection. Table 7 categorizes the primary causes of recurrence of infection with respect to revision type. Of the 60 patients that recurred with infection, 23 patients required amputations with 10 of the amputations resulting from dislocation (Fig. 7).

**Table 7:** Primary cause of recurrence based on type of revision.

Revision Type	Primary Cause of Recurrence							
	Fracture	Draining Sinus	Dislocation	Flap/Soft tissue defect	Wound Dehiscence	Trauma/Fall	Iatrogenic	Total
Knee	2	18	5	6	2	1	1	35
Hip	–	5	11	2	–	–	3	21
Elbow	–	1	–	–	–	–	1	2
Shoulder	–	–	2	–	–	–	–	2

Final analysis of results demonstrated an approximate 88% success rate (440/500). This was compared to a previously published 85% success rate (2000 cases) for the two stage revision used for statistical analysis [8]. Table 8 shows the Fisher exact table with a Fisher exact statistic value is 0.1012. The result is not significant at p ​< ​0.01. Table 9 breaks down success rates based on clinical staging and the corresponding Fisher’s exact test of significance of 0.3902 comparing the Stage III-C-3 success rates with the combined success rate of the other patients with different clinical stages (III–C- 2, III-B-2, III-B-3). Table 10 breaks down flap coverage rate based on revision type.

**Table 8:** Fisher exact comparing results of the one stage with two stage revision^a^.

	Successful	Recurred	Marginal Row Total
One Stage Revision	440	60	500
Two Stage Revision	1700	300	2000
Marginal Column Total	2140	360	2500

a The Fisher exact statistic value is 0.1012. The result is *not* significant at p ​< ​0.01.

**Table 9:** Success rate in candidates for one-stage treatment^a^.

Stage	No. of cases	Success Rate (%)
III-C-3	486	88
III-C-2	2	100
III-B-2	6	100
III-B-3	6	100

Fisher’s exact test of significance ​= ​0.3902. The result is *not* significant at p ​< ​0.01.

a The clinical stages represented were those patients treated by the author from 2005 through 2017 with a minimum follow-up of 2 years. 2 year success rates are shown for each clinical stage.

**Table 10:** Free or local flap coverage.

Total Revision	Flap (%)
Knee	24
Hip	13
Shoulder	75
Elbow	20
Hip-femur-knee	100

Over 97% of PJIs analyzed in this study were classified as Cierny-Mader type IV BLS borderline C- host or McPherson type III-C-3.

## Discussion

As of today, there exists no consensus on definitive techniques for the treatment of PJI. There is agreement, however, regarding the fundamental principles for managing PJI with a one or two-stage revision [13]. These principles involve removal of all hardware, radical debridement which we define as removal of all biofilm infected tissues, then either placement of a stabilized spacer for the joint (two stage) or a definitive reconstruction with antibiotic loaded cement and antibiotic loaded calcium sulfate hemihydrate pellets (one stage). These allow for elution profiles that are log 2–3 above MIC without causing systemic side effects. At these local concentrations, the wound complication rate is 4%. Using the Center for Biofilm Engineering (CBE) drip reactor, the calcium sulfate antibiotic loaded hemihydrate pellets destroyed all biofilm that was surface exposed [13]. A calcium sulfate pellet was beneficial in our implementation of a one-stage procedure because it was bio-absorbable, demonstrated osteoconductive potential, eliminated dead space, and delivered high concentrations of antibiotics locally without adverse systemic toxicity [26]. Compared to other variants, the carrier used was hydrophilic, possessed a physiologic pH, and was synthetically pure. A commonly encountered problem associated with the less pure forms of calcium sulfate carriers has been persistent postoperative wound drainage [7,10].

The one stage, in our opinion, requires a more thorough debridement with a dual setup involving a clean and dirty side. Implant trials were done on the dirty side while the definitive reconstruction was done on the clean side. The advantages to a one stage are reduction in cost greater than 50%, and in our experience, more rapid improvement of function. Eradication of the infection is paramount to avoiding amputations in this scenario. Our results show that comorbidities such as local and systemic compromising factors make the one stage more difficult with all the failures occurring in McPherson stage III-C-3. Unfortunately, we believe this staging system is inadequate for our patient population as almost all of our patients presented as stage III-C-3 by his criteria. We believe a new staging system needs to be adopted for stratification of McPherson III-C-3 patients as many of the referrals had more localized and systemic compromising factors compared to others. Our patient population historically had between 5 and 7 operations for treatment of the PJI prior to referral, accounting for more local and systemic comorbidities.

Two-stage revision protocol adheres to the same principalities for treatment and over the past 3 decades has emerged as the most widely adopted technique for management of chronic infection in total joints. However, this procedure is not without serious disadvantages. As an alternative, single-stage revision avoids some of the drawbacks such as cost and decreased function over a longer period of time inherent in a standard two-stage approach.

The primary goal for surgeons is to implement a therapeutic strategy capable of providing their patients with the best possible outcomes. With this in mind, a one-stage revision of PJI represents an option that gives comparable outcomes to the two-stage revision. By eliminating re-operation, there is a decreased risk of recurrence. We feel that the key to successful outcomes is attributed to the complete removal of biofilm-mediated infection (radical debridement), reversal of amenable comorbidities, and introduction of antimicrobials locally at concentrations to destroy offending pathogens.

While there exists an accepted standard of positive culture identification of organisms in PJI, these standards when applied clinically don’t always yield the expected results. In our cohort, we were only able to recover 34.4% of any infecting organisms. This was due to the fact that the average number of prior surgeries for patients in this study was historically five to seven and that most patients were already on some form of antibiotics. Even the highest recorded organism recovery rate from tissue was only quoted at around 61–65% [43]. In cases where joints present with inflammation, clinicians will often treat with antibiotics and surgical debridement despite the absence of positive cultures. Prosthetic joints can also be infected despite cultures from aspirates and intraoperative samples showing negative results. The reality remains that most culture preparations are inadequate in evaluating culturable elements of biofilm colonies with there being a stark contrast between culture data and modern molecular diagnostic methods. Implementation of next generation 16s DNA sequencing technologies has shown promising results. In a study consisting of 300 patients, subjects were examined using a FISH probe designed to react directly with 16S rRNA of *S. aureus*. Large numbers of *S. aureus* cells were identified for all cohort biofilm microcolonies. Additionally, PCR testing of in vitro samples has shown to be another accurate and efficient method for identifying various microorganisms [4]. Further evaluation and implementation of modern technology along with other novel molecular diagnostic technologies can render problems associated with biofilm culturability obsolete [11,22].

Our review of one-stage revision of infected total joints demonstrates comparable eradication rates to the two-stage revision (88% vs. 85%). Using Fisher’s exact test of significance, the p-value was greater than 0.01 showing that there is no statistical significance between the two surgical protocols. Other studies quote eradication rates for the one stage ranging from 67% to 100% [11,40,42]. A one-stage treatment strategy is more cost-effective and is not associated with some of the physically debilitating complications such as fibrosis, pain, and instability seen with two-stage revision. We believe new strategies are needed in order to improve antibiotic treatment of bacterial biofilm. Novel methods such as laser-induced vapor nanobubbles (VNB) which aim to enhance antibiotic penetration of biofilms should be further studied for in vivo application [41]. With the noted advantages of the one stage coupled with the continued advancement of antibiotic delivery mechanisms and physiologic reconstructions, perhaps treatment with one-stage surgical protocol will become more popular for the treatment of PJI. It is important to note that this type of procedure is best reserved for large referral institutions with multidisciplinary approaches for PJI [13].

There are multiple limitations to this study. Firstly, this study design is retrospective, not prospective, and uses historical controls. In addition, a significant number of the surgical bed cultures were negative. Nonetheless, this can be justified by the referral nature of the practice and the extensive prior treatment with multiple rounds of surgery and antibiotic prior to referral which would limit culture retrieval of infecting organisms. Usually with suppression, there is more of the phenotypic expression of biofilm which can take up to 72 ​h to convert to the dividing planktonic phenotype [22]. With new PCR driven techniques for identifying specific bacteria and bacterial resistance, we believe culture sensitivity in identifying biofilm related hardware infections can be avoided [11]. Advantages of the study are that it was a single surgeon with multiple anatomic sites, minimum 24-month follow-up and equal male/female distribution. Also, the debridement was very radical (it removed all dead bone, reactive and dead soft tissue, and all hardware), similar to a tumor resection. High local levels of local antibiotic were achieved in the periprosthetic environment through the use of an antibiotic loaded calcium sulfate hemihydrate bioabsorbable delivery and antibiotic loaded cement. The benefit of the bioabsorbable antibiotic delivery system is that it can achieve log 2–3 times MIC [13,26]. With use of wafers of this calcium sulfate hemihydrate loaded with 240 ​mg of liquid tobramycin and 500 ​mg of vancomycin in 10 ​cc of the Stimulan pellets in the CBE drip reactor, it completely killed pseudomonas, ERC-1, and MRSA [26].

## Ethics approval

All procedures in this study were in accordance with the 1964 Helsinki Declaration (and its amendments) and the details of the Ethics Committee or institutional review board (IRB) which approved this study in three different hospital systems.

## CRediT authorship contribution statement

**Gerhard E. Maale:** Conceptualization, Methodology, Investigation, Supervision. **John J. Eager:** Data curation, Writing - original draft. **Aniruth Srinivasaraghavan:** Data curation, Writing - original draft, Visualization, Formal analysis. **Daniel Kazemi Mohammadi:** Writing - review & editing. **Nicole Kennard:** Writing - review & editing.

## Declaration of competing interest

The authors declare the following financial interests/personal relationships which may be considered as potential competing interests: Gerhard Maale: Smith and Nephew Royalties; Waldemar LINK Consultant.

John Eager: none
Aniruth Srinivasaraghavan: none
Daniel Mohammadi: none
Nicole Kennard: none

## References

[1] Beuerlein M.J., Mckee M.D. (2010 Mar). Calcium sulfates: what is the evidence?. J Orthop Trauma.

[2] Buchholz H.W., Elson R.A., Engelbrecht E., Lodenkamper H., Rottger J., Siegel A. (1981). Management of deep infection of total hip replacement. J Bone Joint Surg Br.

[3] Cadambi A., Jones R.E., Maale G.E. (1995). A protocol for staged revision of infected total hip and knee arthroplasties: the use of antibiotic-cement implant composites. Int Orthop.

[4] Carter K., Doern C., Jo C.H., Copley L.A. (2016). The clinical usefulness of polymerase chain reaction as a supplemental diagnostic tool in the evaluation and the treatment of children with septic arthritis. Journal of pediatric orthopedics.

[5] Cierny G., Mader J.T., Penninck J.J. (2003). A clinical staging system for adult osteomyelitis. Clin Orthop Relat Res.

[6] Cierny G. (2011 Jan). Surgical treatment of osteomyelitis. Plast Reconstr Surg.

[7] Cierny G., DiPasquale D. (2009). Comparing OsteoSet and stimulan as antibiotic-loaded, calcium sulfate beads in the management of musculoskeletal infection. 19th annual open scientific meeting of the musculoskeletal infection society.

[8] Cierny George, Mader J.T. (1984). Adult chronic osteomyelitis.Orthopedics.

[9] Costerton J.W., Montanaro L., Arciola C.R. (2005). Biofilm in implant infections: its production and regulation. Int J Artif Organs.

[10] Cui Q., Mihalko W.M., Shields J.S., Ries M., Salrli K.J. (2007). Antibiotic-impregnated cement spacers for the treatment of infection associated with total hip or knee arthroplasty. J Bone Joint Surg Am.

[11] Ehrlich G.D. (2012). Culture-negative infections in orthopedic surgery. Springer Series on Biofilms Culture Negative Orthopedic Biofilm Infections.

[12] Fields M.W. (2020). The establishment of the CBE launched biofilms as a field of specialized Research. Biofilms.

[13] Maale Gerhard E., Hsu Wellington K., McLaren Alex C., Springer Bryan D. (2015). Debridement for orthopaedic infection. Let’s discuss surgical site infections, chap. 5, rosemont, IL: American academy of orthopaedic surgeons (AAOS).

[14] Gristina A.G. (1987). Biomaterial-centered infection: microbial adhesion versus tissue integration. Science.

[15] Haddad F.S., Masri B.A., Campbell D., McGraw R.W., Beauchamp C.P., Duncan C.P. (2000). The PROSTALAC functional spacer in two-stage revision for infected knee replacements. Prosthesis of antibiotic-loaded acrylic cement. J Bone Joint Surg Br..

[16] Hall-Stoodley L., Costerton J.W., Stoodley P. (2004). Bacterial biofilms: from the natural environment to infectious diseases. Nat Rev Microbiol.

[17] Hebert C.K., Williams R.E., Levy R.S., Barrack R.L. (1996). Cost of treating an infected total knee replacement. Clin Orthop Relat Res.

[18] Hofmann A.A., Goldberg T., Tanner A.M., Kurtin S.M. (2005). Treatment of infected total knee arthroplasty using an articulating spacer: 2- to 12-year experience. Clin Orthop Relat Res.

[19] Hryhorowicz M., Lipiński D., Zeyland J., Słomski R. (2017). CRISPR/Cas9 immune system as a tool for genome engineering. Arch Immunol Ther Exp.

[20] Insall J.N., Thompson F.M., Brause B.D. (1983). Two-stage reimplantation for the salvage of infected total knee arthroplasty. J Bone Joint Surg [Am].

[21] Jamsen E., Stogiannidis I., Malmivaara A., Pajamaki J., Puolakka T., Konttinen Y.T. (2009). Outcome of prosthethesis exchange for infected knee arthroplasty: the effect of treatment approach. Acta Orthop.

[22] Costerton John William, Post James Christopher, Ehrlich Garth D., Hu Fen Z., Kreft Rachael, Nistico Laura, Kathju Sandeep, Stoodley Paul, Hall-Stoodley Luanne, Maale Gerhard, James Garth, Sotereanos Nick, DeMeo Patrick (2011). New methods for the detection of orthopedic and other biofilm infections. FEMS Immunol Med Microbiol.

[23] Lewis K. (2010). Persister cells. Annu Rev Microbiol.

[24] Lonner J.H., Desai P., Dicesare P.E., Steiner G., Zuckerman J.D. (1996). The reliability of analysis of intraoperative frozen sections for identifying active infection during revision hip or knee arthroplasty. J Bone Joint Surg Am.

[25] Maale G., Eager J., Srinivasaraghavan A. (2019). Evolution of the 2 stage to a 1 stage in the treatment of infected total joint arthroplasties: results of the first 500 cases. 29th annual open scientific meeting of the musculoskeletal infection society.

[26] Maale G.E., Eager J.J., Mohammadi D.K., Calderon F.A. (2020). Elution profiles of synthetic CaSO4 hemihydrate beads loaded with vancomycin and tobramycin. Eur J Drug Metab Pharmacokinet.

[27] Maale G.E., Pascoe H.R., Piercy E.A. (1993). A standardized approach for the treatment of infected total joint arthroplasties by the DFW sarcoma group osteomyelitis protocol; Staged revisions at 2 weeks using antibiotic-cement-implant composites as spacers,. The Journal of Joint Arthroplasty.

[28] Mader J.T., Shirtliff M., Calhoun J.H. (1997). Staging and staging application in osteomyelitis. Clin Infect Dis.

[29] McPherson E.J., Tontz W., Patzakis M., Woodsome C., Holtom P., Norris L., Shufelt C. (1999). Outcome of infected total knee utilizing a staging system for prosthetic joint infection. Am J Orthoped.

[30] Meek R.M., Masri B.A., Dunlop D., Garbuz D.S., Greidanus N.V., McGraw R., Duncan C.P. (2003). Patient satisfaction and functional status after treatment of infection at the site of a total knee arthroplasty with use of the PROSTALAC articulating spacer. J Bone Joint Surg Am.

[31] Nett M.P., Long W.J., Della Valle C.J. (2014). Diagnosis and treatment of infected total joint arthoplasty. World Clin Orthoped.

[32] Pangaud Corentin (2019). Outcome of single-stage versus two-stage exchange for revision knee arthroplasty for chronic periprosthetic infection. EFORT Open Reviews.

[33] Rand J.A., Bryan R.S., Morrey B.F., Westholm F. (1986). Management of infected total knee arthroplasty. Clin Orthop.

[34] Sanchez-Sotelol Berry DJ., Hanssen A.D. (2009). Mid-term to long-term follow-up of stated reimplantation for infected hip arthroplasty. Clin Orthop Relat Res.

[35] Signore, Alberto (2019). Consensus document for the diagnosis of prosthetic joint infections: a joint paper by the EANM, EBJIS, and ESR (with ESCMID endorsement). Eur J Nucl Med Mol Imag.

[36] Simmons T.D., Stern S.H. (1996). Diagnosis and management of the infected total knee arthroplasty. Am J Knee Surg.

[37] Simpson A.H., Deakin M. (2001). Latham JM.Chronic osteomyelitis: the effect of the extent of surgical resection on infection free survival. J Bone Joint Surg Br..

[38] Stevens C.M., Tetsworth K.D., Calhoun J.H., Mader J.T. (2005). An articulated antibiotic spacer used for infected total knee arthroplasty: a comparative in vitro elution study of Simplex and Palacos bone cements. J Orthop Res.

[39] Subramanian S. (2020). Microsystems for biofilm characterization and sensing – a. Review. Biofilm.

[40] Tande Aaron J., Patel Robin (2014). Prosthetic joint infection. Clinical microbiology reviews vol.

[41] Teirlinck E. (2019). Laser-induced vapor nanobubbles improve diffusion in biofilms of antimicrobial agents for wound care. Biofilms.

[42] Thakrar R.R. (2019). Indications for a single-stage exchange arthroplasty for chronic prosthetic joint infection. The Bone & Joint Journal, 101-B, no. 1_Supple_A.

[43] Trampuz A., Piper K.E., Jacobson M.J., Hanssen A.D., Unni K.K., Osmon D.R., Mandrekar J.N., Cockerill F.R., Steckelberg J.M., Greenleaf J.F., Patel R. (2007). Sonication of removed hip and knee prostheses for diagnosis of infection. N. Engl. J Med.

[44] Windsor R.E., Insall J.N., Urs W.K., Miller D.V., Brause B.D. (1990). Two-stage reimplantation for the salvage of total knee arthroplasty complicated by infection: further follow-up and refinement of indications. J Bone Joint Surg Am.

[45] Wolf C.F., Gu N.Y., Doctor J.N., Manner P.A., Leopold S.S. (2011). Comparison of one and two-stage revision of total hip arthroplasty complicated by infection: a markov expected-utility decision analysis. J Bone Joint Surg Am.

[46] Woods J. (2012). Development and application of a polymicrobial, in vitro, wound biofilm model. Journal of applied microbiology.

